# Implications for megathrust earthquakes and tsunamis from seismic gaps south of Java Indonesia

**DOI:** 10.1038/s41598-020-72142-z

**Published:** 2020-09-17

**Authors:** S. Widiyantoro, E. Gunawan, A. Muhari, N. Rawlinson, J. Mori, N. R. Hanifa, S. Susilo, P. Supendi, H. A. Shiddiqi, A. D. Nugraha, H. E. Putra

**Affiliations:** 1 Global Geophysics Research Group, Faculty of Mining and Petroleum Engineering, Institut Teknologi Bandung, Bandung, 40132 Indonesia; 2 Faculty of Engineering, Maranatha Christian University, Bandung, 40164 Indonesia; 3 National Disaster Management Authority, Jakarta, 13120 Indonesia; 4 Department of Earth Sciences – Bullard Labs, University of Cambridge, Cambridge, CB3 0EZ UK; 5 Disaster Prevention Research Institute, Kyoto University, Uji, 611-0011 Japan; 6 National Center for Earthquake Studies, Jalan Turangga No. 3-5, Bandung, 40263 Indonesia; 7 Badan Informasi Geospasial, Cibinong, 16911 Indonesia; 8 Agency for Meteorology, Climatology and Geophysics, Bandung, 40161 Indonesia; 9 Department of Earth Science, University of Bergen, Allègaten 41, 5007 Bergen, Norway; 10 PT. Reasuransi Maipark, Multivision Tower, Menteng Atas, Jakarta, 12960 Indonesia

**Keywords:** Seismology, Natural hazards, Geophysics, Tectonics

## Abstract

Relocation of earthquakes recorded by the agency for meteorology, climatology and geophysics (BMKG) in Indonesia and inversions of global positioning system (GPS) data reveal clear seismic gaps to the south of the island of Java. These gaps may be related to potential sources of future megathrust earthquakes in the region. To assess the expected inundation hazard, tsunami modeling was conducted based on several scenarios involving large tsunamigenic earthquakes generated by ruptures along segments of the megathrust south of Java. The worst-case scenario, in which the two megathrust segments spanning Java rupture simultaneously, shows that tsunami heights can reach ~ 20 m and ~ 12 m on the south coast of West and East Java, respectively, with an average maximum height of 4.5 m along the entire south coast of Java. These results support recent calls for a strengthening of the existing Indonesian Tsunami Early Warning System (InaTEWS), especially in Java, the most densely populated island in Indonesia.

## Introduction

**Figure 1:**
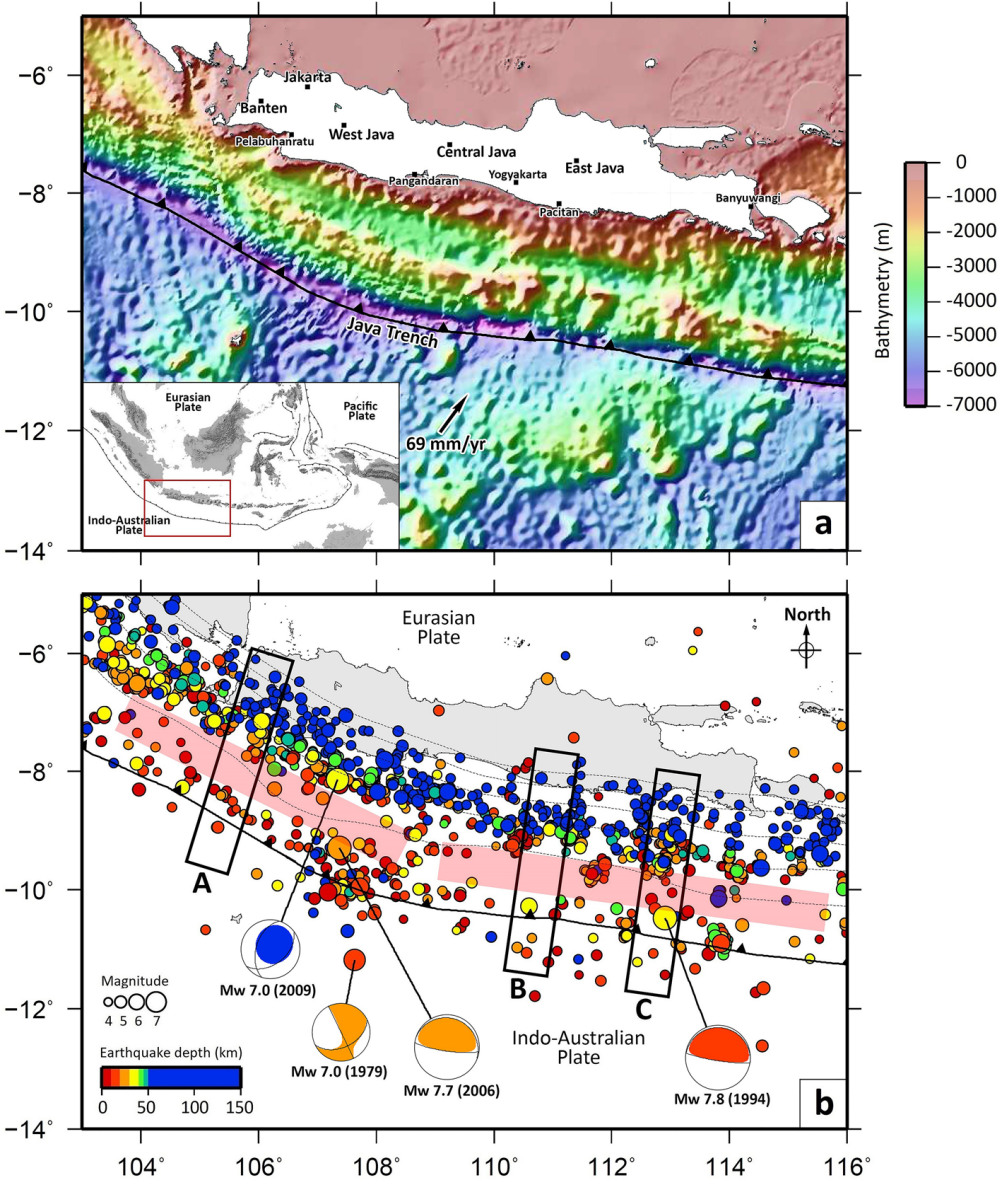
Regional setting and distribution of epicenters. (**a**) Map of the study area. Plate motion is from Altamimi et al.^5^. The bathymetry data were taken from ETOPO1^6^. Inset shows the location of the study area (red rectangle) with respect to southeast Asia. (**b**) Distribution of epicenters of relocated earthquakes with magnitude ≥ 4.0. The earthquake data (2009–2018) were taken from BMKG. The focal mechanisms of events with M > 7.0 are plotted at ISC-EHB locations and are taken from the Global Centroid Moment Tensor (gCMT) solution catalog (https://www.globalcmt.org); (cf. Supplementary Fig. 2, i.e., all ISC-EHB locations from 1964 to 2016). Note that focal mechanisms are coloured according to gCMT depths, which are not always consistent with the ISC-EHB depths e.g. the 1994 Java earthquake, which is most likely shallow. Regions that lack seismicity (as approximately indicated by the red shaded areas on the map) are interpreted as seismic gaps. Black rectangles (**A**–**C**) indicate the location of vertical cross sections shown in Fig. 2. We intentionally compress the colour mapping so that all events below 50 km depth are dark blue such that it is more straight forwards to distinguish upper plate crust events from downdip interface events, and events in the oceanic plate from updip interface events.

Seismic gaps off the southwest coast of Sumatra, which pose a risk for generating future megathrust events, have been studied in detail (e.g., Refs.^1–4^). On the other hand, seismic gaps further southeast along the Sunda Arc adjacent to Java, the main island of Indonesia with a total population of more than 150 million people, have been less intensively studied. The regions along the southern coast of Java, e.g., Pelabuhan Ratu, Pangandaran, Pacitan, and Banyuwangi (see Fig. 1a), have grown rapidly in recent times and are prone to large earthquakes and their associated tsunamis, which can be devastating. In 1994 and 2006, tsunamigenic earthquakes of magnitude < 8 occurred near Banyuwangi (Mw 7.8), East Java^7^ and Pangandaran (Mw 7.7), Central Java^8^, respectively (Fig. 1), and the accompanying tsunamis killed a combined total of nearly a thousand people.

South of Java, Jurassic-age seafloor with a thick sediment cover is subducting beneath the edge of the Sundaland margin at the Java Trench (see e.g., Ref.^9^). The absence of recent great earthquakes may indicate that even more powerful tsunamigenic events along the south coast of Java are a potential threat. If this is true, then the release of effective early warnings needs to be a high priority, since most people living in high tsunami risk areas will have little time to evacuate. However, the presence of a seismic gap does not necessarily imply the accumulation of elastic strain, since slow slip events could be responsible for ongoing energy release^10,11^. While there is no evidence for slow slip along the Java Trench, this may be due to a lack of observations, such as could be acquired from seafloor geodetic investigations^12^. Nevertheless, while slow slip cannot be ruled out, recent studies^13^ have found evidence of tsunamigenic deposits along the coastline facing the Java Trench, which point to the occurrence of historic megathurst events.

In this study, we use new data taken from the Indonesian Tsunami Early Warning System (InaTEWS) catalog reported by BMKG, together with data from the International Seismological Centre (ISC) catalog, to investigate the potential for megathrust earthquakes and consequent tsunamis south of Java. From this combined dataset, we extract P- and S-wave arrival-times from 436 seismic stations at local, regional, and teleseismic distances in the period from April 2009 to November 2018 (Supplementary Fig. 1a). We limited the earthquake data to those events that have an azimuthal gap of less than 210°, following^14^, and at least 10 P- and S-phase arrival times. Using this dataset, we were able to accurately relocate a total of 1898 earthquakes with Mw ≥ 4.0 in the study region. The relocation was achieved using the teleseismic double-difference technique that is part of the teletomoDD method introduced by Pesicek et al.^15^, which also permits local and regional recordings of earthquakes to be included.

Besides the seismic data analysis, we also inverted 6 years of GPS data from 37 stations in Central and East Java to investigate slip deficits that can lead to future earthquakes. These results were combined with those of a previous study of West Java in order to generate a composite model of slip deficit rate for the whole of Java. The source areas and expected slip inferred from the GPS data inversion results are then used for numerical modeling of tsunami heights via finite difference solution of the long wave equation. Three different rupture scenarios are tested, with a focus on maximum wave height along the south coast of Java.

## Results

**Figure 2:**
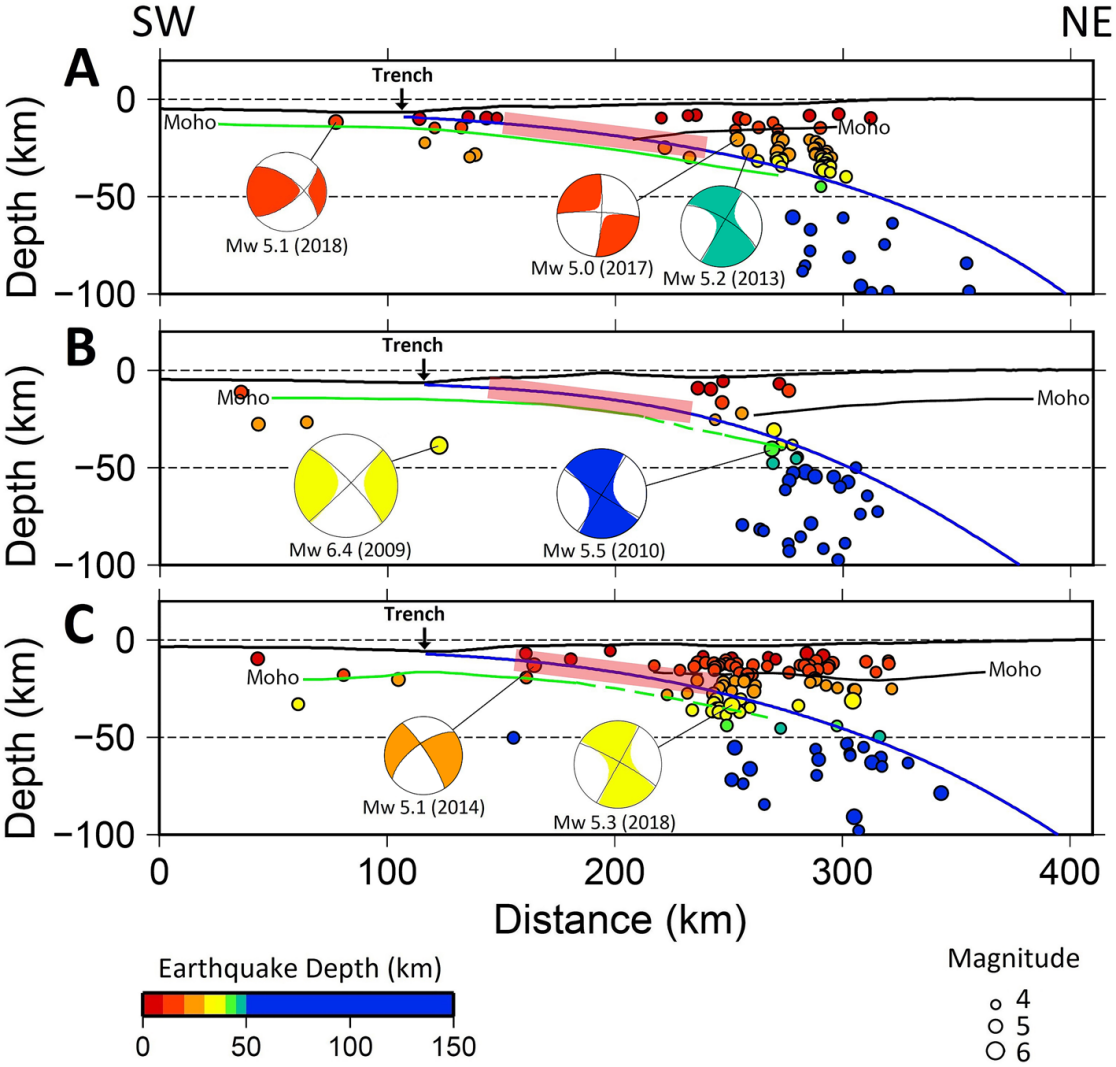
Cross sections of relocated earthquakes with magnitude ≥ 4.0. The locations of cross sections **A**–**C** are shown in Fig. 1b. The focal mechanisms of events were taken from the Global Centroid Moment Tensor solution catalog (https://www.globalcmt.org). Blue lines depict the plate interface of the subducted Indo-Australian Plate according to the Slab2.0 model^18^, while green and black lines depict the oceanic and continental Moho depths, respectively (taken from^58–60^). The thick red lines denote the approximate locations of seismic gaps, and are drawn to be consistent with Fig. 1b. Again, we compress the colour mapping so that all events below 50 km are dark blue as in Fig. 1b. Note that focal mechanisms are coloured in the same way as Fig. 1b.

Our earthquake relocation results show clear elongated zones, located between the coast of Java and the Java Trench, which lack seismicity and hence are identified as seismic gaps (Figs. 1b and 2 ). The approximate location of these gaps is highlighted by the red rectangles in Figs. 1b and 2 (see Supplementary Fig. 2 for a comparison plot that also includes all ISC-EHB events of Mw ≥ 4.0 from 1964 to 2016^16^). These gaps are symptomatic of subduction zones which hold significant potential for great earthquakes; elsewhere, such as parts of offshore Sumatra (see Supplementary Fig. 3), where recent seismicity has filled the seismic gaps, large earthquakes are not expected. If it is assumed that the gap corresponds to a lack of earthquakes on the plate interface^17^ of the subducting slab, as described by the Slab2.0 model^18^, then the “locked” region of the subduction interface extends to a depth of between 20 and 30 km (Fig. 2), close to the base of the upper plate crust, which would be consistent with a typical seismogenic zone for large subduction zone earthquakes.

The epicentral shifts of relocated events are in general perpendicular (north–south direction) to the Java Trench (Supplementary Fig. 1b and c), which may be caused by the stations being mainly distributed to the north on Java^15,19^. Similarly, since all seismometers are placed on the surface above the earthquakes, there are also shifts in the vertical direction (Supplementary Fig. 4). Following the hypocenter relocations, there is a significant reduction in travel-time residuals as depicted by the histograms of relative residuals (Supplementary Fig. 5), which indicates a better travel time fit for the new locations. Statistical uncertainties of the hypocentral parameters, which are also produced by the relocation, are shown in Supplementary Fig. 6.

**Figure 3:**
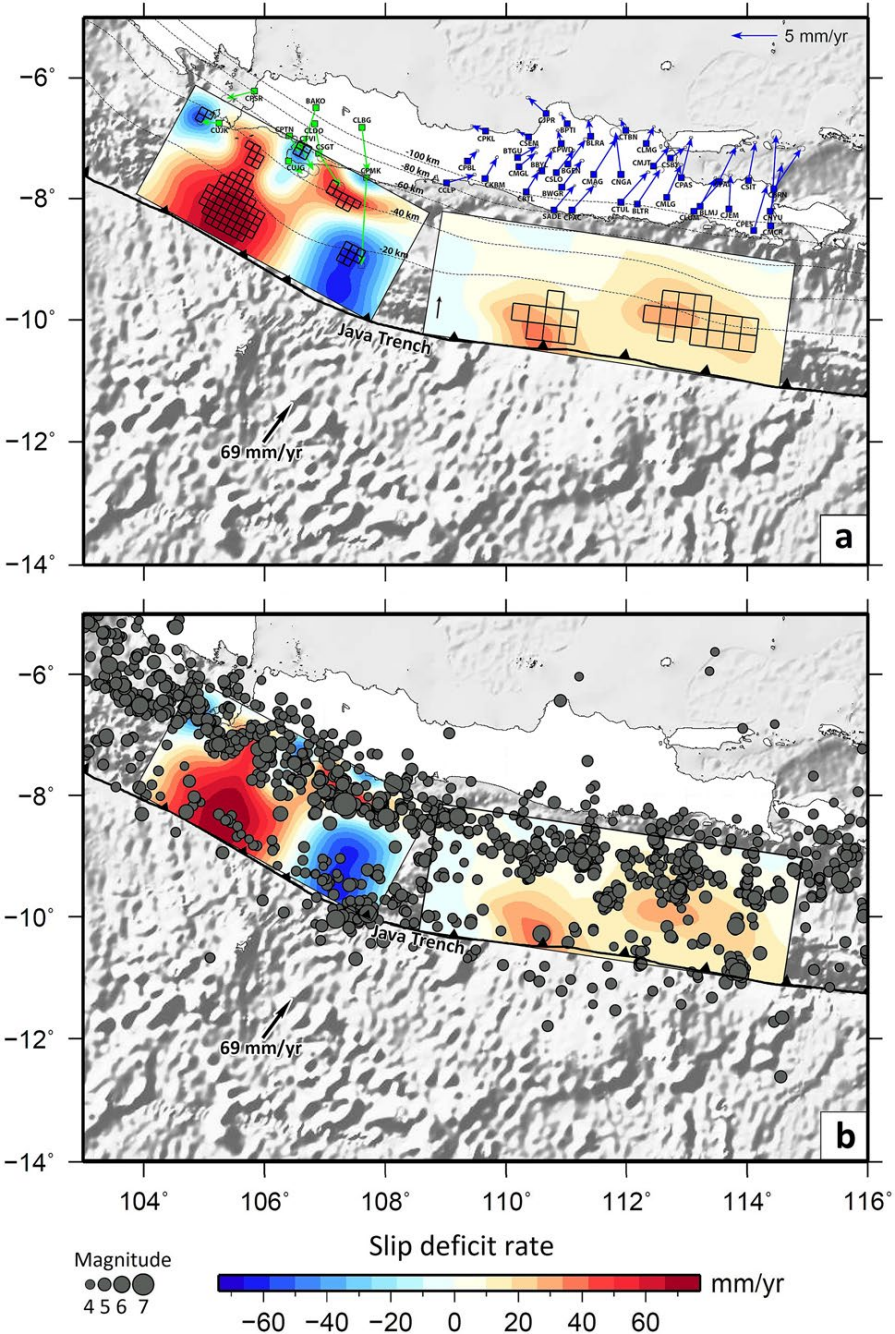
Interplate coupling models. (**a**) Slip deficit/excess along the Java Trench derived from GPS data that reflects the segmentation of the megathrust to the south of Java. Left: model derived by Hanifa et al.^20^; Right: model produced by this study. Black squares are superimposed to indicate where the estimated uncertainty is lower than the absolute value of the predicted slip deficit rate. See Supplementary Fig. 7b for an uncertainty map based on the posterior covariance matrix. Colour scale depicts the estimated slip-deficit rate (red) and the slip-excess rate/afterslip (blue). Dashed lines are contours of the top of the slab from the Slab 2.0 model^18^. Green squares depict the locations of GPS stations used by Hanifa et al.^20^ to constrain the model south of West Java, and the blue squares denote the locations of GPS stations used in this study to produce the model south of Central and East Java. Arrows show GPS velocities relative to the Sundaland block reference frame. Blue arrows represent the GPS velocities derived in this study after removing the postseismic deformation of the 2006 Java tsunami earthquake. Green arrows are GPS velocities taken from Hanifa et al.^20^. Note that most vectors are aligned in the direction of current plate motion. This could be an indication of strong seismic coupling to the south of the study area. The red areas of slip deficit indicate areas with increased potential for a great earthquake. These regions may rupture in individual events or together in the worst-case scenario. Plate motion is taken from the ITRF2014 model by Altamimi et al.^5^. (**b**) Same as (**a**) but with the epicenter distribution shown in Fig. 1b overlaid on the slip deficit model.

By comparing the observed deformation field with long term plate motion models, the GPS data inversion results are able to reveal the present day strain accumulation process, which likely reflects a longer term build-up of strain energy (Fig. 3). Where the observed GPS deformation is less than the plate motion (slip deficit), the areas are inferred to be likely sources of future earthquakes. The area of strongest slip deficit is south of West Java (Fig. 3), which may be a potential source for a megathrust earthquake^20^. The inversion of GPS data from Central and East Java undertaken in this study shows similar features, i.e., two zones with slip deficits, albeit of lower magnitude compared to the west, which is unsurprising considering the differences in GPS velocities between the two regions (Fig. 3). The first high-slip deficit zone is located on the shallower part of the fault off the south coast of Central Java. The second high-slip deficit region is in the deeper part of the megathrust seismogenic zone off the south coast of East Java.

The assumption that the areas of large slip deficit will correspond to areas of large seismic slip during future large earthquakes may turn out to be an over-simplification. Examples from other regions reveal a complicated relationship between geodetic measurements and slip during earthquakes^21^. However, we still feel that it is a useful exercise to investigate this scenario, since it does provide a means to estimate potential tsunami heights in the event of a future great earthquake. The approach and assumptions we adopt are similar to those used for the Nankai trough, where areas of strong geodetic coupling are assumed to be areas of large slip during earthquakes^22^.

If we adopt the above assumption, then the area of high slip deficit may rupture separately or together during an earthquake. The area of the deficit zone south of West Java is equivalent to a Mw 8.9 earthquake, assuming a return period of 400 years (consistent with Harris et al.^13^ and Okal^23^). For the same return period, the area of high slip deficit in Central and East Java is equivalent to a Mw 8.8 earthquake, whereas if both areas rupture in a single earthquake, it would produce a Mw 9.1 event.

**Figure 4:**
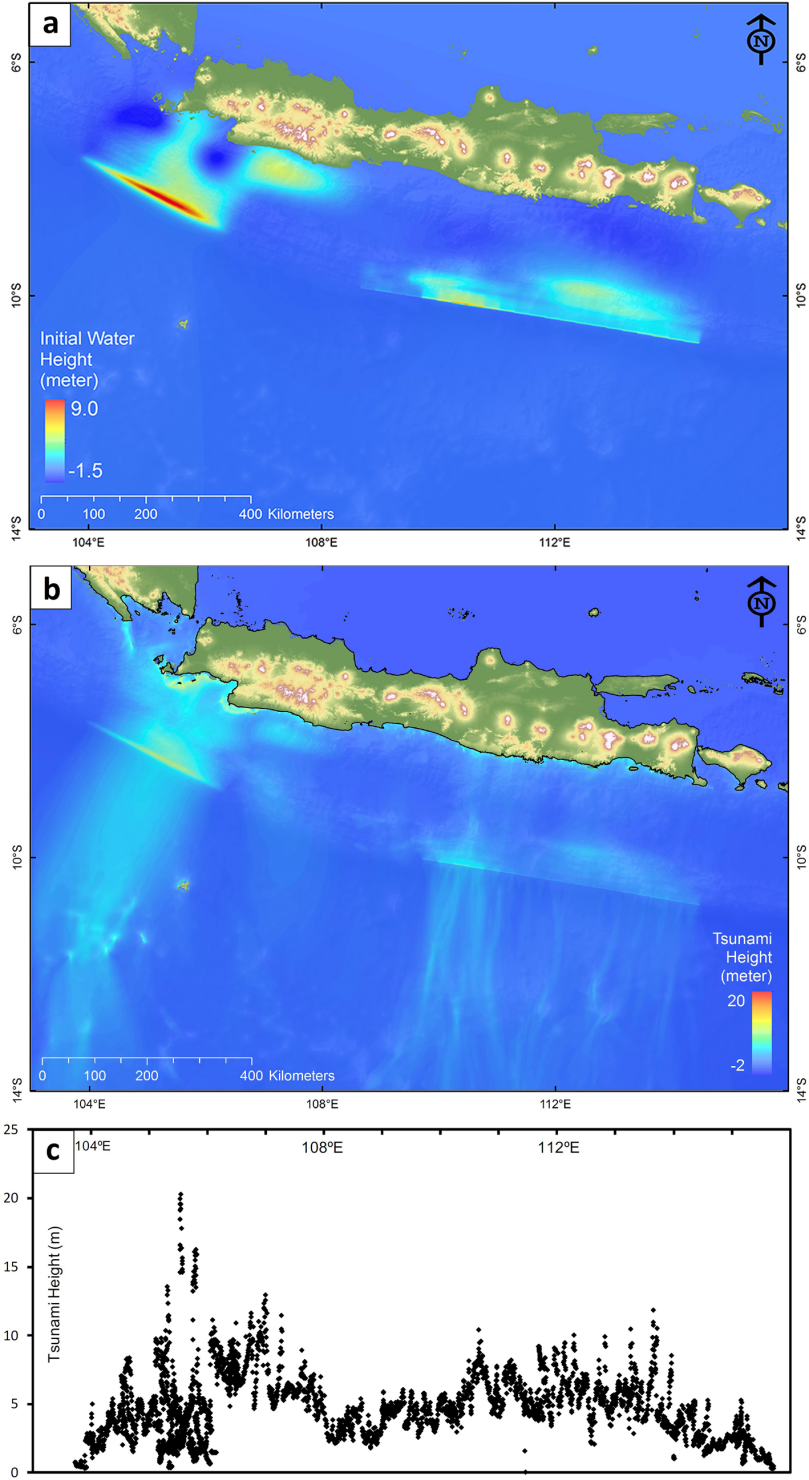
Modeled tsunami heights along the south coast of Java. This model is based on the worst-case scenario in which the modeled tsunami sources off the south coast of Java rupture at the same time. (**a**) Combined modeled tsunami sources off the south coast of Java from Scenarios 1 and 2. (**b**) Maximum tsunami height throughout model region over duration of simulation. (**c**) Maximum tsunami height along the south coast of Java.

To assess the potential tsunami heights along the south coast of Java we conducted tsunami modeling using several different scenarios. Hypothetical megathrust segments are available from the National Center for Earthquake Studies of Indonesia (see Supplementary Fig. 9), but in this study we used megathrust segments from our GPS data inversion, which we regard as more realistic since we estimated the expected amount of slip. Here, we carried out tsunami modeling with three different megathrust scenarios: (1) western Java segment only (Mw 8.9), (2) eastern Java segment, i.e., to the south of Central and East Java, only (Mw 8.8), and (3) western and eastern Java segments (Mw 9.1). The results of Scenarios 1 and 2, along with initial water heights for all three scenarios, are presented in Supplementary Figs. 10–12. The worst-case scenario (Scenario 3) with a return period of 400 years can generate a giant earthquake of Mw 9.1 and a very large tsunami with a maximum height of 20.2 m near the small islands to the south of Banten, the westernmost province of Java, at ~ 105.5°E (Fig. 4). We note that the tsunami heights can be even higher than modeled when slumping occurs, as has been suggested in the case of the 2018 M 7.5 Palu strike-slip earthquake in Sulawesi, East Indonesia, where submarine landslides may have contributed to tsunami generation^24^.

## Discussion

Okal^23^ presented a detailed study of the 1921, 1937 and 1943 intraplate earthquakes south of Java, and found that the Java subduction zone lacks large interplate thrust events for the entire era of instrumental seismicity. However, he also remarked that this does not exclude the possibility that larger earthquakes occur over recurrence periods longer than the instrumental history, thereby leaving open the possibility that megathrust events will occur along the Java Trench. The results of our study, based on the seismic and GPS data, show that in the scenario we develop, there is high potential for megathrust events off the southern coast of Java, as suggested by the observed seismic gaps depicted in Figs. 1b and 2. The locations of the seismic gaps appear broadly consistent with the areas of slip deficit along the Java Trench as determined from interplate coupling models derived from GPS data (Fig. 3).

As acknowledged previously, seismic gaps may be caused by slow slip events that do not manifest seismically^10,11^, and the recent < 8 Mw events in 1994 and 2006 may represent the upper limit of earthquake size along the Java Trench. However, in light of recent megathrust events such as the 2004 Sumatera and 2011 Tohoku earthquakes, determining maximum moment release based on historic recordings or models can produce conservative estimates, and all subduction zones of sufficient length should be regarded as possible hosts of megathrust earthquakes^24^. In the case of the Java Trench, its potential in this regard is supported by a recent study^13^ that found tsunami deposits at many sites along the coastline facing the Java Trench, which lead the authors to conclude that megathrust earthquakes occur with a return period of approximately 500 years. Consequently, we believe that our models represent a plausible scenario, but acknowledge that the potential for slow slip and complexities in the relationship between slip deficit and earthquake rupture may also play a role in determining the likelihood of future great earthquakes.

The results of our tsunami modeling, performed using the tsunami sources derived from the GPS data, i.e., the interplate coupling models, show that the worst-case scenario (Scenario 3), where the tsunami sources off the south coast of Java all rupture at the same time, produces a tsunami height up to 20.2 m and 11.7 m in West and East Java, respectively. These maximum tsunami heights are similar to those obtained from Scenarios 1 and 2, but the combined tsunami sources increase the average tsunami height along the south coast of Java to ~ 4.5 m (Fig. 4). We note that the larger value in the west may in part be a consequence of the slip deficit modelling undertaken by Hanifa et al.^20^, which did not account for the effects of post-seismic deformation following the 2006 Mw7.8 earthquake, and hence should be regarded as an upper limit. Nevertheless, even if maximum wave heights in West Java are similar to those in East Java, they would pose a risk to densely populated cities in the region such as Pelabuhan Ratu.

Our multidisciplinary study, which includes seismic and geodetic data analyses and tsunami wave height modelling, clearly reveal the presence of seismic gaps off the southern coast of Java that could be the source of future great earthquakes with tsunamigenic potential. Although this is only one possible scenario, with mitigating circumstances such as slow slip potentially reducing the likelihood and severity of such an event, it nevertheless seems prudent to support recent calls^13,25^ for additional submarine and sea level instruments for the relatively sparse InaTEWS network in south Java, to help protect the many people living in the coastal areas.

## Materials and methods

### Data sources

The earthquake data used in this study were taken from the BMKG and ISC data catalogues. The GPS data were obtained from the Geospatial Information Agency of Indonesia and the Ministry of Agriculture and Spatial Planning/Land Administration Agency as part of the Indonesian Continuously Operating Reference Stations network. Plate motion and the bathymetry data were taken from Altamimi et al.^5^ and ETOPO1^6^, respectively.

### Double difference earthquake relocation

We used the teletomoDD algorithm, which is an extension of the double difference tomography method^26^ to teleseismic distances^15^. Following Pesicek et al.^15^, in this study we kept the initial seismic velocity model fixed and used the relocation capabilities only. Travel times were calculated using a 3D regional seismic velocity model of the Indonesian region with a grid size of 1° × 1°^27^ and the ak135 global 1D model^28^ for regions outside Indonesia. We employed the pseudo-bending ray tracing technique^29^ adapted for use in a spherical coordinate system by Koketsu and Sekine^30^. This ray tracing method allows for the prediction of local, regional, and teleseismic P- and S-wave arrival-time data, which enabled us to incorporate both BMKG data and data recorded by regional and global networks. The initial number of earthquakes available in our catalogue is 2312. However, prior to relocation, we implement selection criteria to eliminate poorly constrained events. The selection criteria are: (1) the events have at least 10 P- and S-phase arrival times, and (2) the azimuthal gaps are less than 210° for local/regional stations in the Indonesian network. A total of 2046 events passed the selection process and were subsequently relocated using the double difference approach. A total of 20 iterations of the double difference algorithm were applied, which yielded 1,898 well located events (see Figs. 1b and 2) and 148 poorly located events that were discarded from the final dataset.

The distribution of seismic stations used in this study, the epicentral shifts following event relocation, and a Rose diagram highlighting trends in the epicentral shifts are shown in Supplementary Fig. 1. In Supplementary Fig. 2 we plot the hypocenters of all events of Mw > 4.0 from the ISC-EHB catalogue from 1964 to 2016^16^, which illustrates that the seismic gaps we identify south of Java are still present for this much larger dataset. Supplementary Fig. 4 displays a comparison between the BMKG hypocenters and those resulting from the teletomoDD relocation, the hypocenter shifts, as well as along-dip-distance of hypocentral shift as a function of azimuth. Supplementary Fig. 5 shows histograms of relative residuals before and after relocation, respectively. Here, we note that the histograms were constructed using the relative residual data of event pairs as required in the teletomoDD technique^15^ and not the absolute residual data of each event.

To estimate the location uncertainties associated with teletomoDD hypocenter results, we performed a jackknife resampling analysis^31,32^. Following Tichelaar and Ruff^33^ and Halpaap et al.^34^, we created a number of resampled datasets by removing a fixed number of our input data. In our case, we created 100 datasets by randomly deleting 10% of the differential travel-times data. We re-ran the hypocenter relocation using these datasets and computed the standard deviations of the earthquake locations. The average horizontal and depth uncertainties are 3.3 and 5.3 km, respectively (Supplementary Fig. 6).

### GPS data processing

We used GPS data from 37 stations (see Fig. 3) located in central and eastern Java from 2008 to 2014, of which 20 are international GNSS stations. GAMIT/GLOBK software^35,36^ was used to analyze the daily solutions of the GPS recordings. The processing strategy for the GPS data follows the approach described in our previous study^37^. Final solutions of the GPS analysis are in the International Terrestrial Reference Frame (ITRF) 2008^38^.

Gunawan et al.^39^ pointed out that the mainshock of the 2012 Mw 8.6 Indian Ocean earthquake affected global tectonic displacements as far away as Java. Thus, the GPS daily solutions in this study are corrected for the coseismic displacements of the 2012 earthquake. Daily solutions were then transformed into the Sundaland block reference frame based on the transformation parameters of Altamimi et al.^5^. From these GPS data, we generated velocity estimates by applying linear regression at each GPS station.

In the western part of the study area, an Mw 7.8 tsunamogenic earthquake occurred in 2006^40^. Previous studies have suggested that the area around the mainshock continued to deform following the rupture due to postseismic deformation (e.g., Refs.^41,42^). In most cases, the mechanism of postseismic deformation associated with viscoelastic deformation generates a broader deformation area than afterslip^43–45^.

Modelling studies^37,46^ have suggested that the postseismic deformation of the 2006 earthquake involved a long duration of afterslip and a viscoelastic relaxation that affected Java^12^. We removed the effect of the postseismic deformation of the 2006 earthquake at each GPS station by employing afterslip and viscoelastic modelling as proposed by Gunawan et al.^37^. Thus, the ‘corrected’ velocities at each GPS station become the final data used for further analysis. Using the corrected velocity at each GPS station, we can then conduct slip deficit/excess modeling.

Figure 3 shows a map of GPS velocities for Central and East Java derived in this study after removing the postseismic deformation of the 2006 Java earthquake, together with GPS velocities for West Java derived by Hanifa et al.^20^, which have not been corrected for postseismic deformation. Most vectors point in the direction of current plate motion, indicating strong seismic coupling to the south of Java.

### Slip deficit/excess modeling

Using the corrected velocity at each GPS station, we analyzed the slip deficit/excess in the central-eastern part of Java using the ‘backslip’ concept to characterise interseismic deformation at subduction zones^47^. In our study area, tectonically stable locations for analyzing slip deficit/excess are not available. Thus, we used an analysis of baseline length changes to calculate the slip deficit/excess of the fault plane along the Java Trench^20,48,49^.

In our analysis, we constructed a fault plane of 660 km length and 210 km width, which was discretized into 30 km × 30 km fault patches. Based on the Slab2.0 model for megathrust geometry^18^, we employed a dip angle of 16° from the surface to 52 km depth, and a dip angle of 34° for greater depths. The fault strike is assumed to be 278°, while the rake is 270°.

We designed a network of baselines by connecting pairs of GPS stations in this region using the same approach adopted by Hanifa et al.^20^. From the 37 continuous GPS stations, we produced 83 baselines. Using the ‘corrected’ velocities, we calculated the baseline rate changes. In our inversion analysis, we generated Green’s functions for each baseline using an elastic half-space model^50^. Since all other parameters are defined, the only unknown parameters are the magnitude of slip deficit/excess. In our slip inversion procedure, we employed a priori information in the form of smooth spatial variations with free boundary conditions along the trench and no-slip conditions for the other three fault plane edges (east, west, and north). The slip distribution was then estimated using Akaike’s Bayesian Information Criterion (ABIC)^51–53^. Supplementary Fig. 7a shows the variation of ABIC as a function of the hyperparameter (a dimensionless quantity that controls the relative weighting between fitting the data and the prior smoothness constraints), and the location of the best-fit model.

Using GPS data located in the western part of Java, Hanifa et al.^20^ estimate slip deficit/excess off the southwest coast of Java. In this study, we combine their findings and our new findings in a single plot (Fig. 3a). The combined results show a significant slip deficit/excess distribution along the Java Trench, which can be compared to the distribution of seismicity (see Fig. 3b). In Fig. 3a we superimpose black squares in regions where the absolute value of the slip deficit rate exceeds the model uncertainty. In the case of the eastern segment, this information is derived from the posterior covariance matrix, the diagonal elements of which are plotted in Supplementary Fig. 7b; for the western segment, the corresponding information is supplied by the study of Hanifa et al.^20^. The difference between data observations derived from GPS data and predictions from the slip deficit model are plotted in two ways—as velocity vectors with respect to reference stations in Supplementary Fig. 7c and as baseline change rates in Supplementary Fig. 8.

Based on the above results, the primary regions of slip deficit appear to be robust. We note that combining the GPS data from Hanifa et al.^20^ with the GPS data we processed to produce a single slip deficit model may result in potential inconsistencies since we have removed the effect of the postseismic deformation of the 2006 earthquake at each GPS station by employing afterslip and viscoelastic modelling as proposed by Gunawan et al.^37^, whereas they did not attempt to account for the effects of this event. Indeed, Hanifa et al.^20^ suggest that southward motion of station CPMK (see Fig. 3) is evidence of ongoing afterslip related to this Mw 7.8 earthquake, although whether it occurs inside the mainshock rupture area or in the adjacent downdip area cannot be determined. The reduced amplitudes of the anomalies on the eastern section compared to the western section may in part be a consequence of not accounting for this afterslip.

In terms of spatial resolution, Hanifa et al.^20^ use fault patches of size 12.5 × 12.5 km, which is considerably smaller than ours, but synthetic recovery tests demonstrate that at depths between 30 and 70 km, resolution is ~ 60 × 60 km or better, whereas at depths less than 30 km, it increases to as much as 250 km along strike, and 100 km down dip. A similar pattern of model resolution would also apply to the central-eastern segment recovered in this study.

### Tsunami modeling

Based on the slip deficit/excess modeling results from the GPS data, we modeled the tsunami height and inundation based on several scenarios using the TUNAMI modelling code of Imamura^54^ and Imamura et al.^55^. We prepared a set of bathymetric and topographic data to construct a rectangular grid of points in latitude (5° to 14°S) and longitude (103° to 116°E) (Supplementary Fig. 10) based on the General Bathymetric Chart of the Oceans (GEBCO) 15 arc-second interval datasets (https://www.gebco.net^56^). The model domain, which is transformed to Cartesian coordinates, has 3120 and 2160 nodes in the x (longitude) and y (latitude) directions, respectively, with an equal grid spacing of 463 m. The vertical wall boundary condition is used at the coast, which does not allow for the transfer of momentum flux. This is a common boundary condition for tsunami modelling, which prevents on-shore inundation^54^. We used the long wave equation of Imamura^54^, which was solved using finite differences, to model tsunami propagation from three different source scenarios with various slip distributions. The first scenario is defined by 720 segments in West Java (Scenario 1 with an estimated magnitude of Mw 8.9), the second by 154 segments in East Java (Scenario 2, with an estimated magnitude of Mw 8.8) and the third a combination of scenarios 1 and 2 with an estimated magnitude of Mw 9.1, which is referred to as Scenario 3. The various slip distribution sources were modeled using an elastic deformation model^50^ for each segment. It was assumed that all segments ruptured simultaneously without any consideration of the rupture speed or finite rise time (Fig. 4). The model was run for 5 h to obtain maximum tsunami height along the south coast of Java.

The results of tsunami modeling based on slip deficit/excess results from the GPS data described above are presented in Supplementary Figs. 11 and 12. The resulting tsunami heights along the south coast of Java from Scenario 1 show that the maximum tsunami height is likely to occur in the region between 105° and 106°E, i.e., at the small islands located ~ 11 to 15 km from the nearest coast line where tsunami height can reach up to 20.2 m. This scenario yields an average tsunami height of 3.23 m along the south coast of Java (Supplementary Fig. 11). Based on Scenario 2, a maximum tsunami height of 11.7 m can occur at 113.65°E along the south coast of East Java. The broad rupture zones in this region have resulted in roughly similar peak tsunami heights along the coast between 110°E and 114°E compared to further west, but with a lower average tsunami height of 2.43 m (Supplementary Fig. 12). Wave-height uncertainty is not straight forward to estimate, but our results are largely consistent with those from Horspool et al.^57^, who undertook a probabilistic tsunami hazard assessment of Indonesia, in which they use a different finite difference code, source rupture model and bathymetry (30-arc second model from GEBCO). While our model is more detailed, the basic pattern of peak wave-heights along the south coast of Java is similar, with the largest amplitudes occurring in the west and an average maximum wave-height along the entire coast of between 4 and 5 m.

## Supplementary information

Supplementary Figures.

## Data Availability

Relocated events from the BMKG catalogue that were used in this study can be downloaded from https://doi.org/10.5281/zenodo.3749015, and the slip deficit model can be downloaded from https://doi.org/10.5281/zenodo.3935744. Maximum tsunami wave heights determined from our modelling can be downloaded from https://doi.org/10.5281/zenodo.3750914. ISC-EHB arrival time and event location data are freely available from https://www.isc.ac.uk. GPS data is available by request from the Indonesian Geospatial Information Agency (BIG)—see https://www.big.go.id/en.
