# Supplementary material for: Implications for megathrust earthquakes and tsunamis from seismic gaps south of Java Indonesia
Source: Sci Rep. 2020 Sep 17;10:15274. doi: 10.1038/s41598-020-72142-z (PMC7499206; doi:10.1038/s41598-020-72142-z)
Supplement: Supplementary file 1 — Supplementary Figures. [file 41598_2020_72142_MOESM1_ESM.pdf]

Supplementary information to the manuscript

Implications for megathrust earthquakes and tsunamis from seismic gaps south of Java Indonesia

By S. Widiyantoro, E. Gunawan, A. Muhari, N. Rawlinson*, J. Mori, N. R. Hanifa, S. Susilo,

P. Supendi, H. A. Shiddiqi, A. D. Nugraha, H. E. Putra

*Corresponding author, nr441@cam.ac.uk

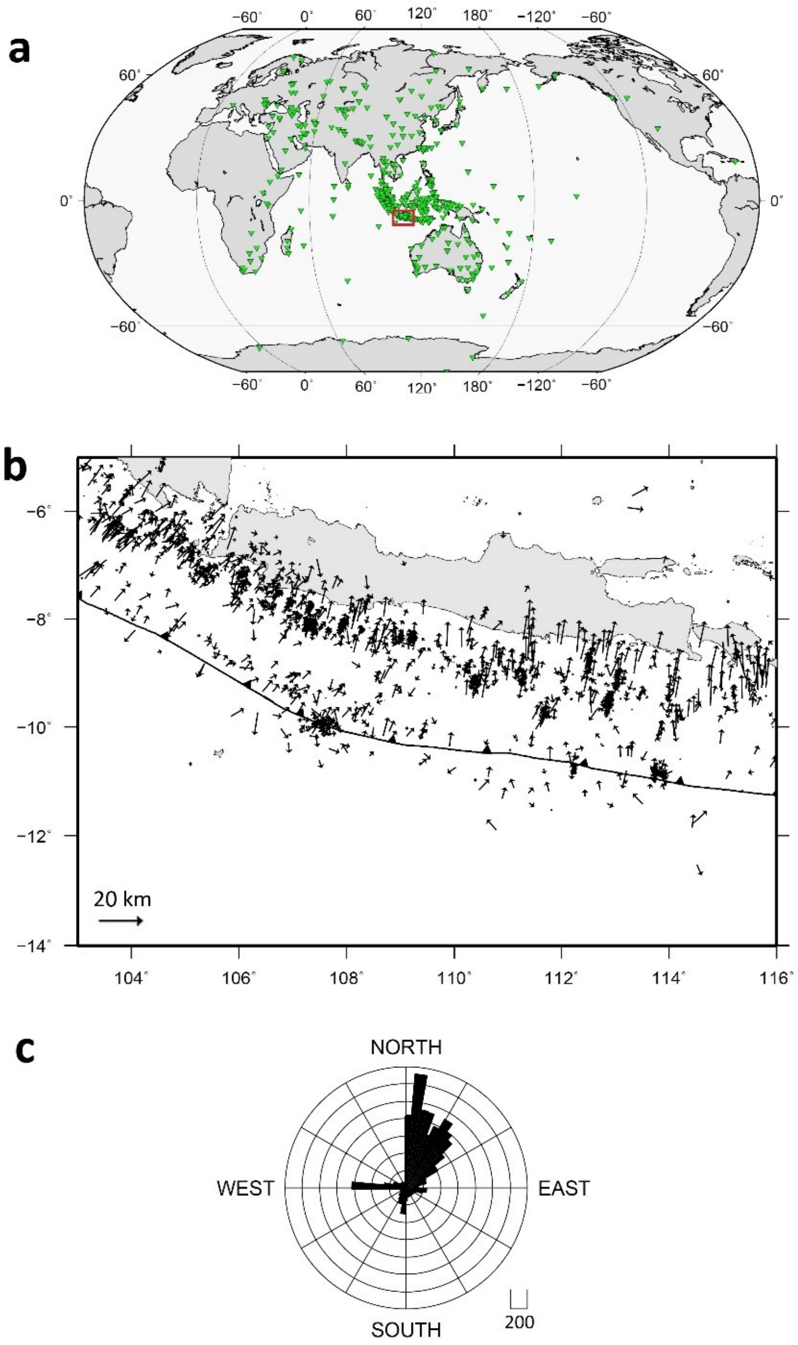

Supplementary Figure 1. *(a) Distribution of seismic stations used by BMKG to locate events including those from outside Indonesia; the red box depicts the study area shown in (b). (b) Epicenter shifts of the teletomoDD locations relative to the initial locations taken from the*

BMKG catalog; the mean epicenter shift displacement is 9.67 km. (c) Rose diagram showing the dominant direction of relocation shifts for 5° azimuth bins. Notice that most of the shifts are in the direction of relative plate motion, nearly perpendicular to the axis of the Java Trench. We note that the original hypocenters from BMKG, before the relocation, also exhibit gaps similar to what we get from the relocated events.

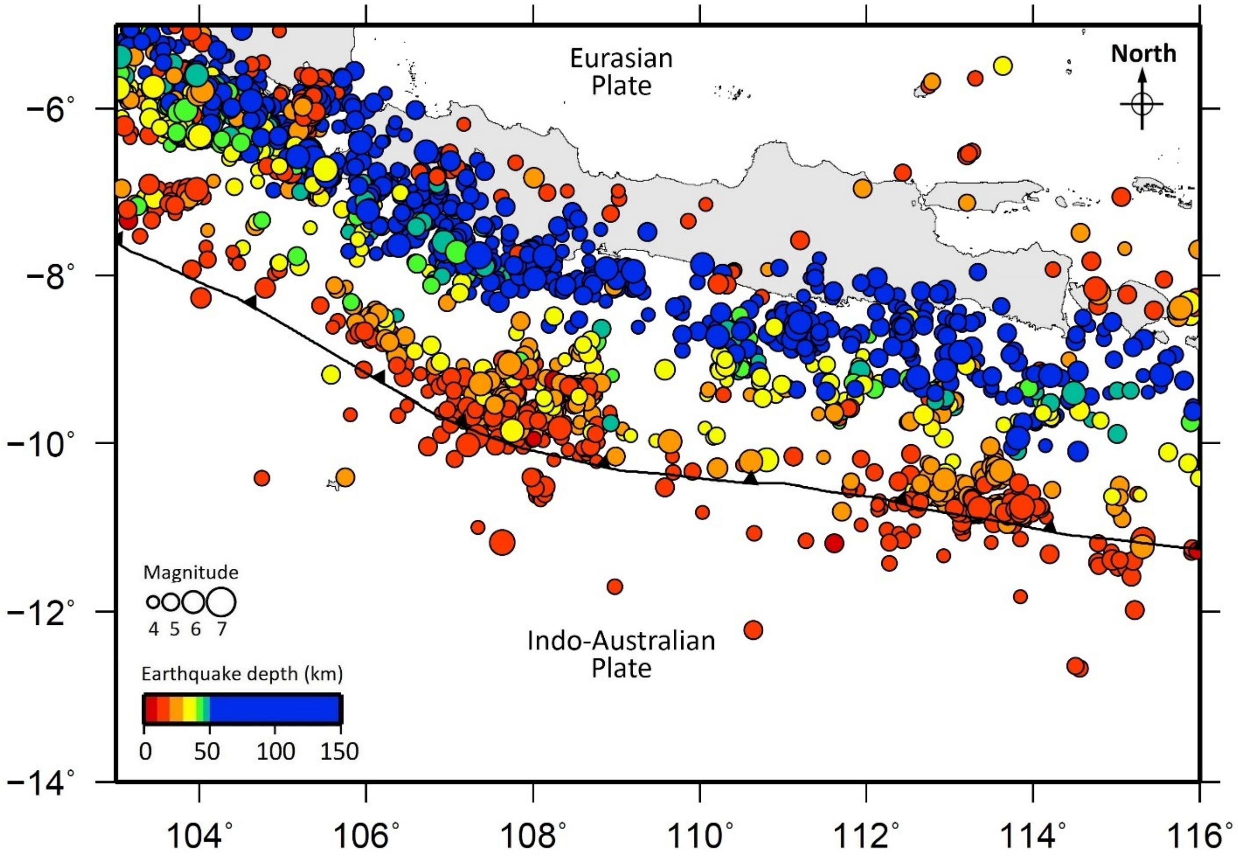

Supplementary Figure 2. *Distribution of epicenters of relocated earthquakes with ISC-EHB locations from 1964 to 2016 [16]. Note that the seismicity gaps identified in Fig. 1 (b) of the main manuscript are still present in this much larger dataset.*

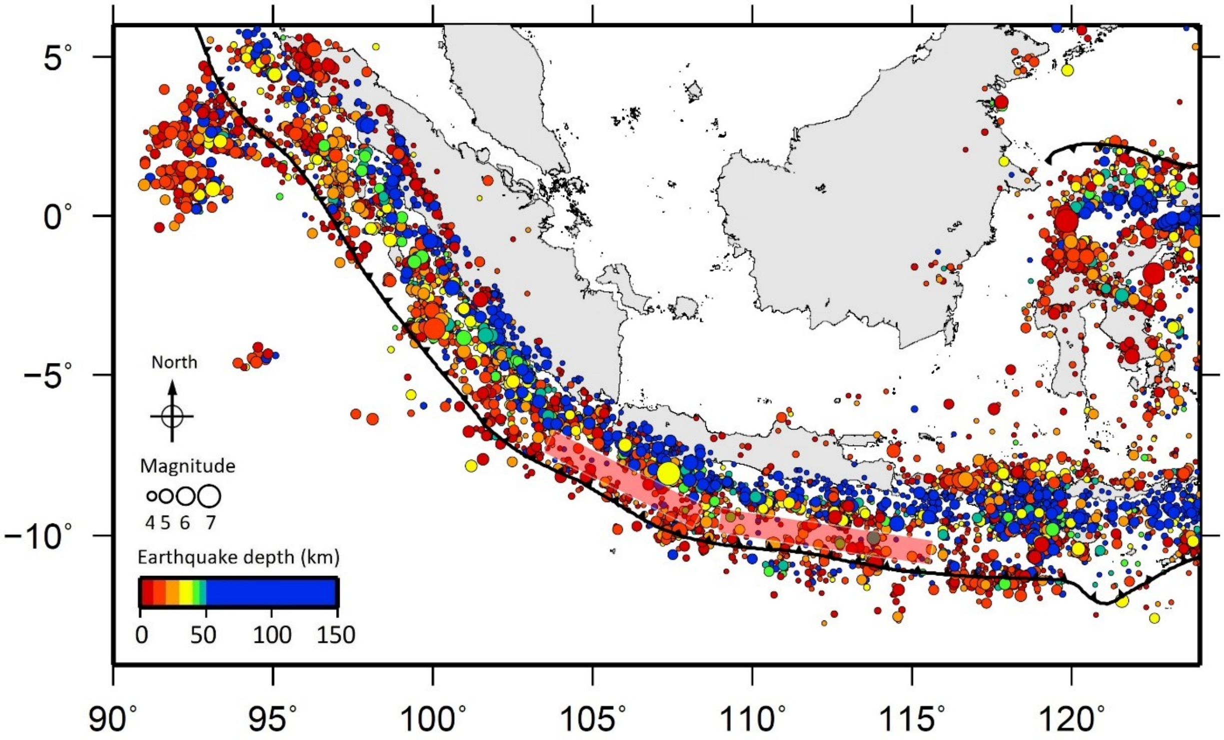

Supplementary Figure 3. *Distribution of epicenters of relocated earthquakes from 2009 to 2018 for M > 4.0 and events shallower than 150 km as shown in Fig. 1b, but for the Sunda arc. The red rectangles show the interpreted seismic gaps south of Java where there are relatively fewer earthquakes between the trench and deeper subduction events as also shown in Fig. 1b.*

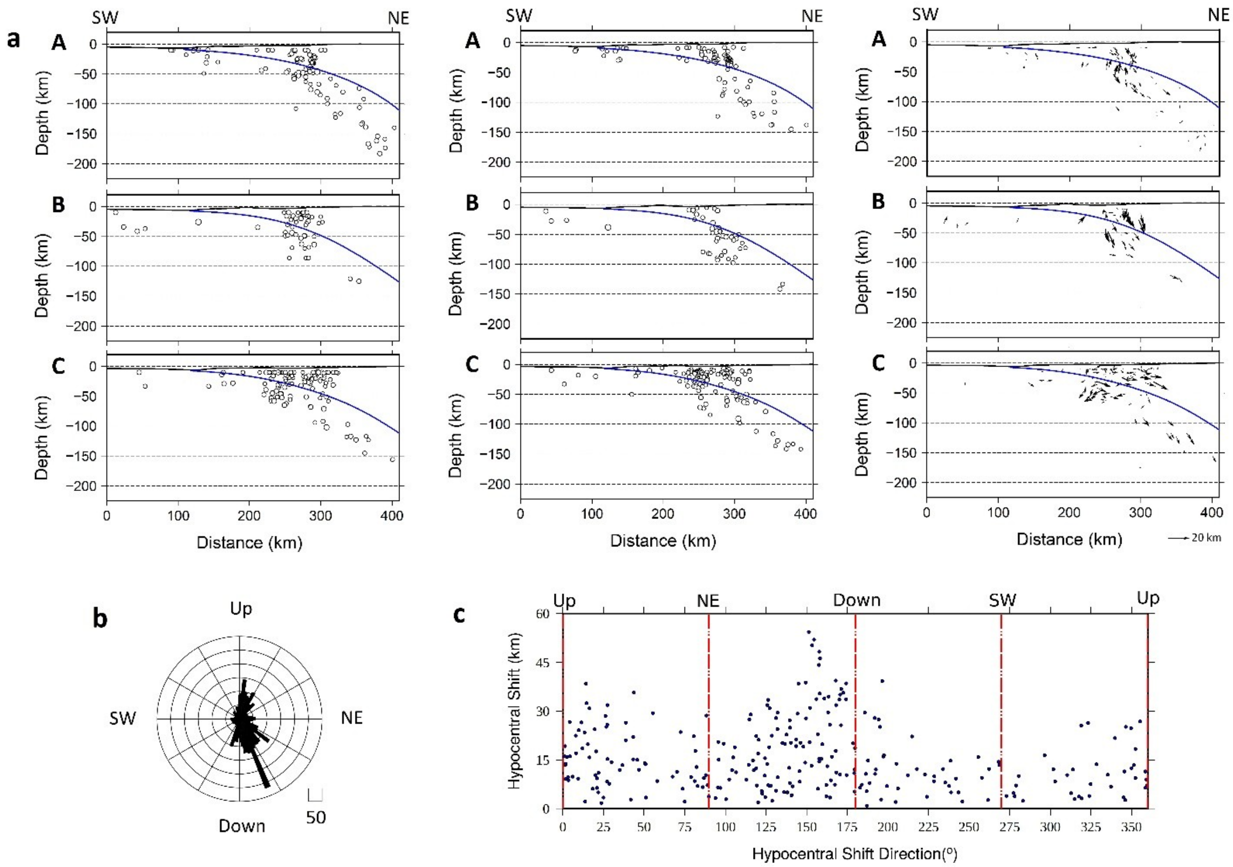

***Supplementary Figure 4**. (a) Comparison of cross sections of hypocenters from the BMKG catalog and the teletomoDD relocation result, and the hypocenter shifts. Blue lines depict the upper surface of the subducting Indo-Australian slab according to the Slab 2 model [18]. The location of each cross section (A-C) is shown in Fig. 1b. The mean focal depth shift is 12.05 km. (b) Rose diagram showing the directions of the shifts of hypocenters from a distance up to 40 km on both sides of the cross sections, which are primarily in the direction of plate subduction. (c) Along-dip-distance of hypocentral shift as a function of azimuth.*

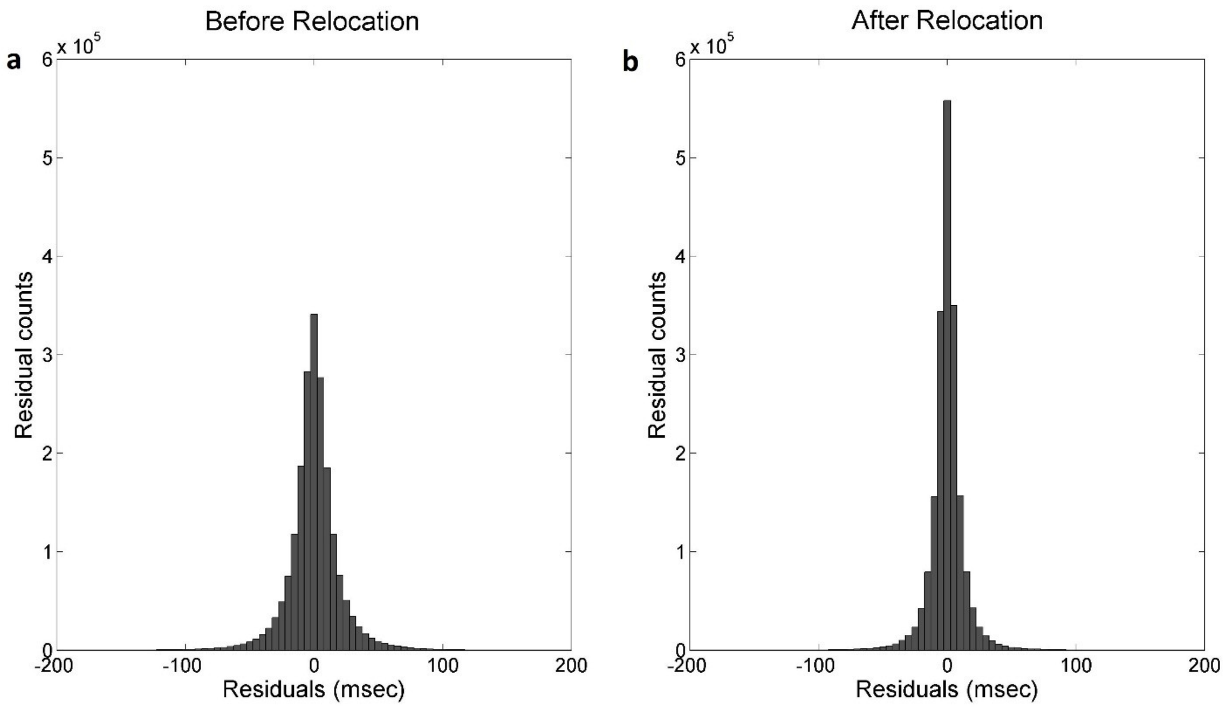

Supplementary Figure 5. *Histograms of relative residuals of event pairs. (a) Before relocation. (b) After relocation using the teletomoDD technique.*

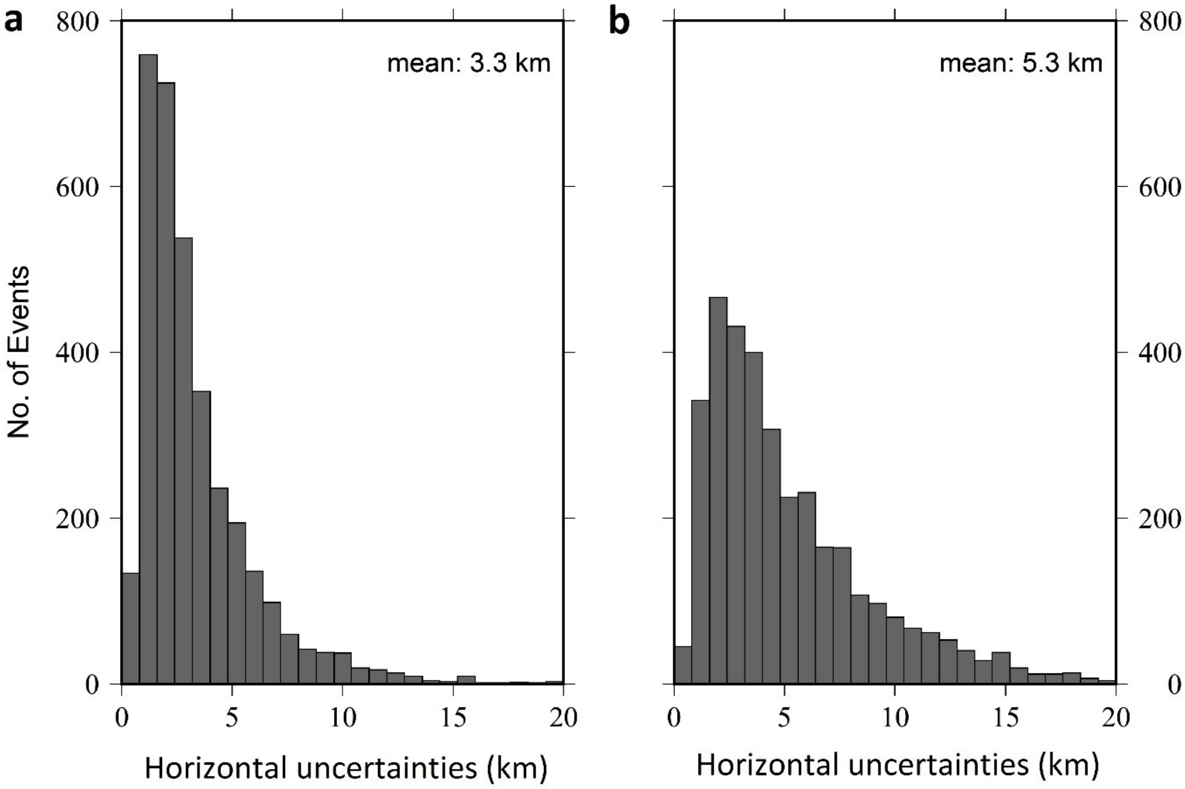

Supplementary Figure 6. *Histograms of epicenter and focal depth uncertainties for earthquake locations in the study region. (a) Uncertainties in the horizontal direction. (b) Uncertainties in the vertical direction.*

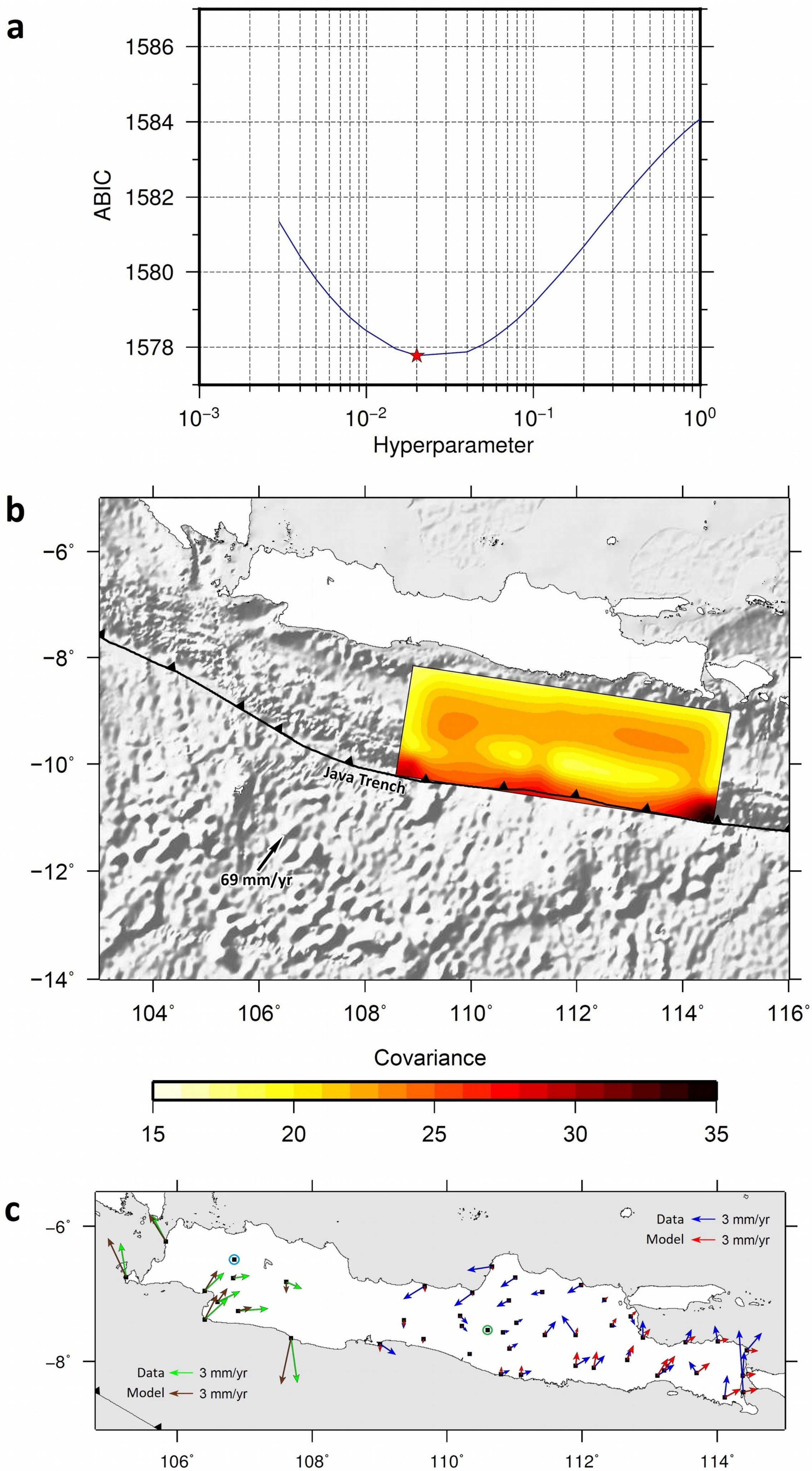
a
ABIC
1586
1584
1582
1580
1578
10^{-3}
10^{-2}
10^{-1}
10^{0}
Hyperparameter
b
-6°
-8°
-10°
-12°
-14°
104°
106°
108°
110°
112°
114°
116°
Java Trench
69 mm/yr
Covariance
15
20
25
30
35
c
-6°
-8°
106°
108°
110°
112°
114°
Data 3 mm/yr
Model 3 mm/yr
Data 3 mm/yr
Model 3 mm/yr

Supplementary Figure 7. *(a) Hyperparameter as a function of ABIC with best-fit parameter shown by the red star. (b) Diagonal elements of the covariance matrix determined as a result of the inversion analysis. (c) Velocity vector comparison for the West/East Java model between data observations (green/blue arrows) and model predictions (black/red arrows), with an average error of 0.6 mm, with respect to station BAKO/BBYL (blue/green circled square). Error is a measure of the difference between data and model velocity vectors at each station. The larger misfit between the observations and model predictions in easternmost Java is likely due to the presence of two active local faults – the Wongsorejo Fault and Kendeng Fault along Madura strait - the effects of which are not removed prior to the inversion procedure.*

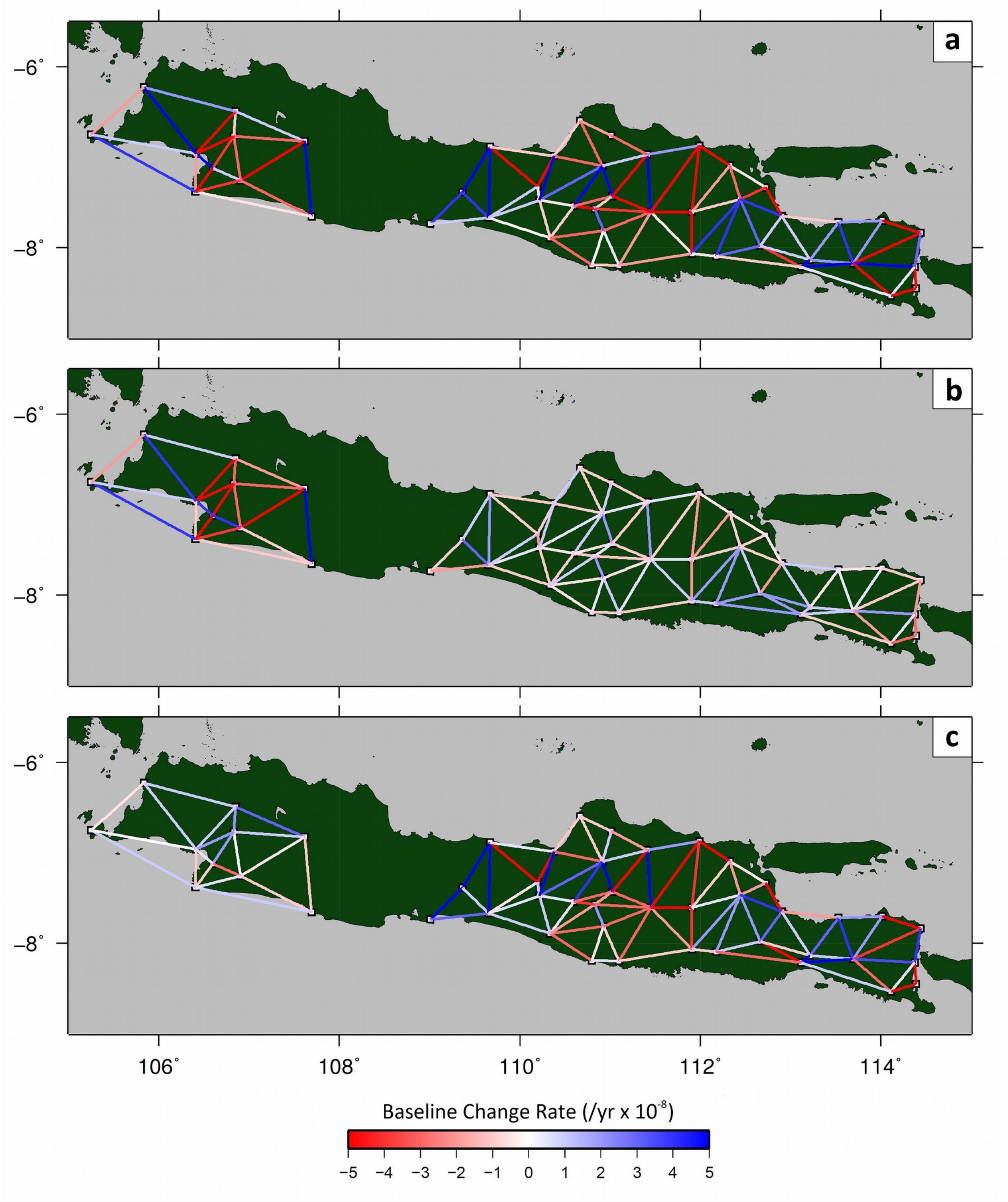

Supplementary Figure 8: (a) Observed and (b) predicted baseline change rates determined from the GPS data and slip deficit models (see Fig. 3) respectively. (c) Difference between observed and predicted baseline change rates. cf. Supplementary Fig. 7c.

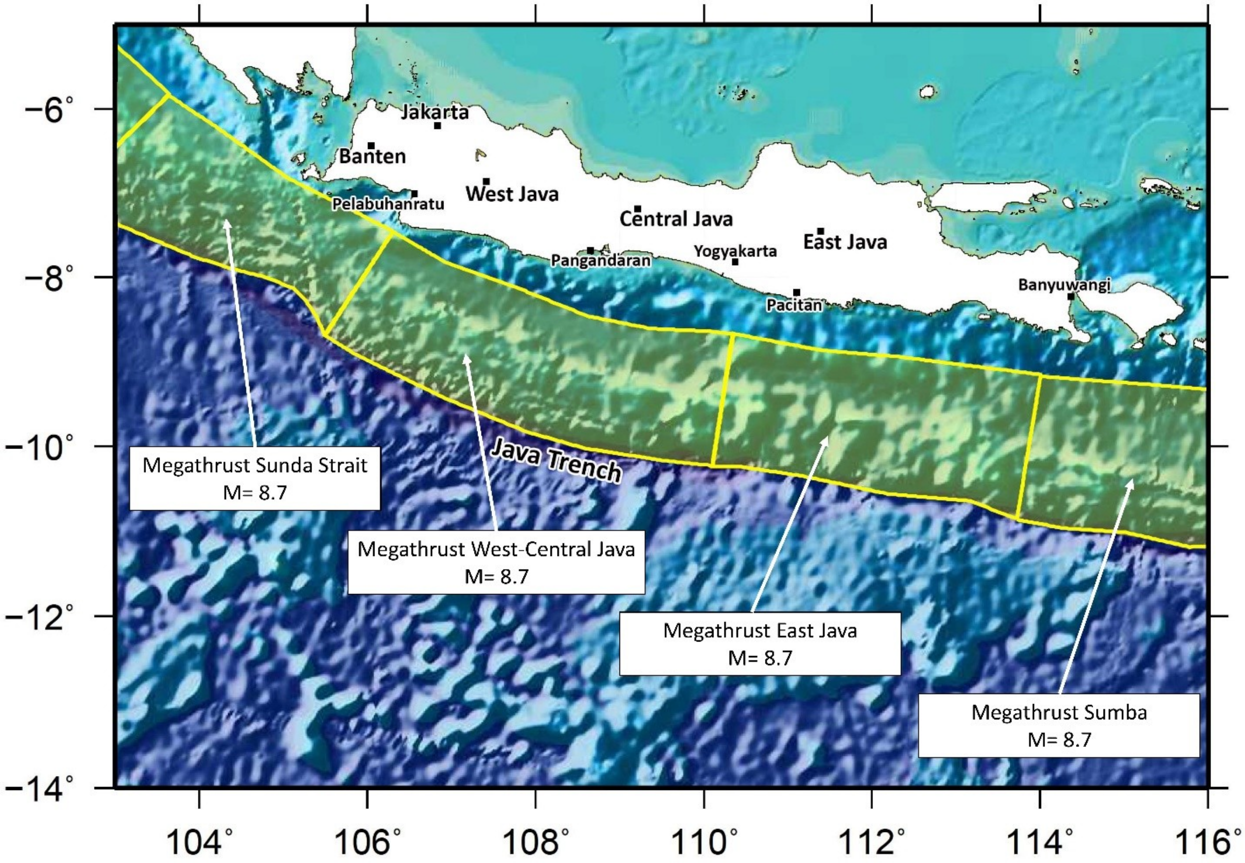

***Supplementary Figure 9**. Subduction zone sources. The hypothetical segmentation of the megathrust along Java and their seismic parameters based on Irsyam et al. [62].*

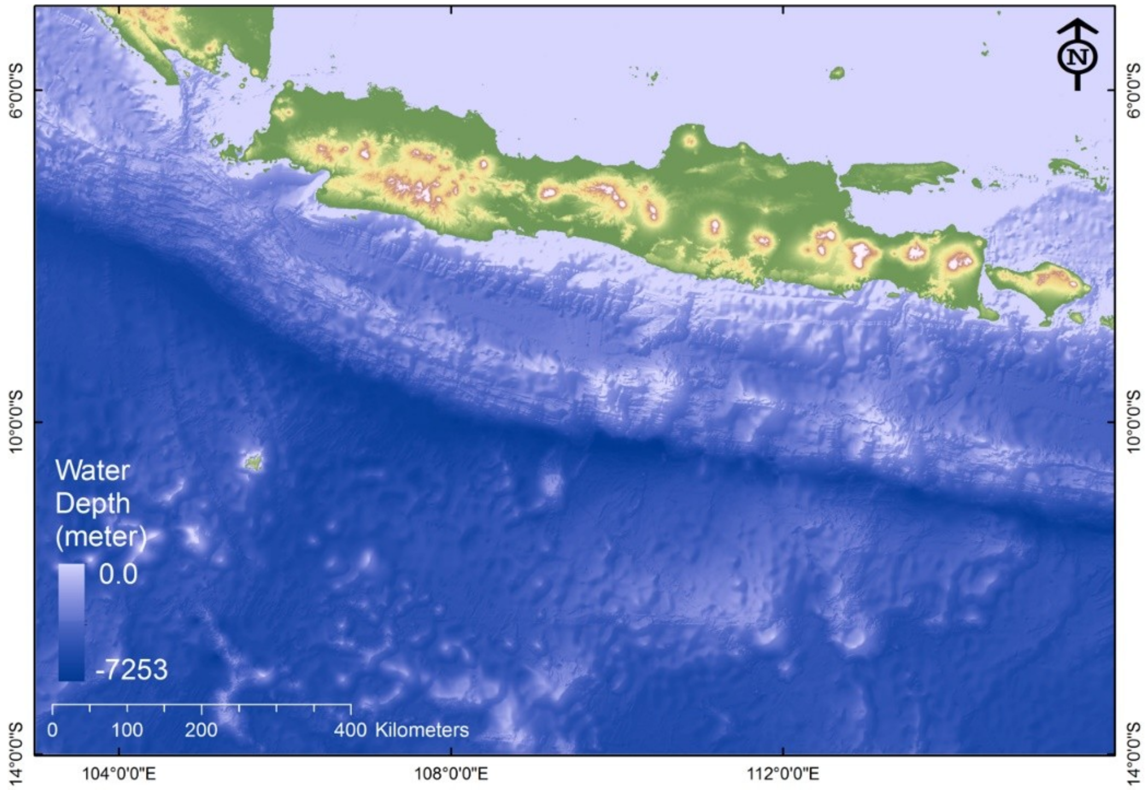

Supplementary Figure 10. *The model domain for tsunami simulation. The water depth data were taken from the General Bathymetric Chart of the Oceans 15 arc seconds datasets (https://www.gebco.net).*

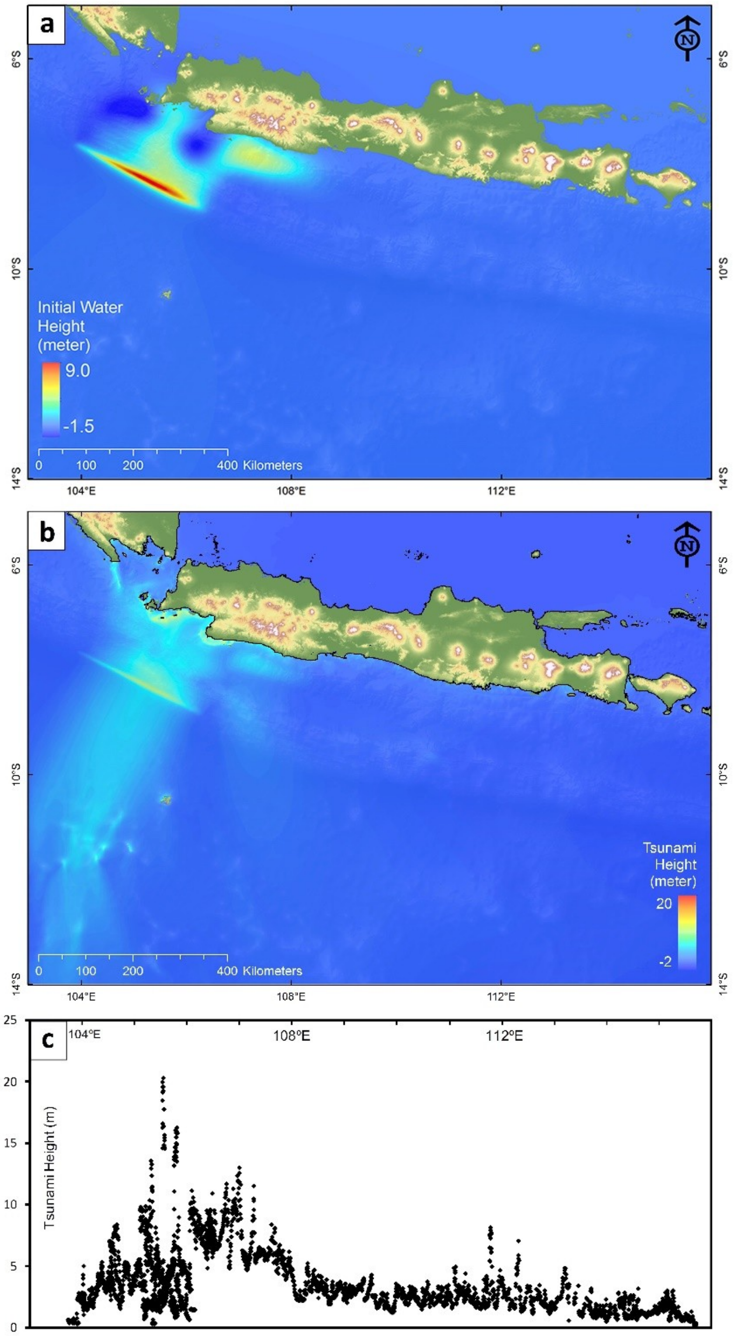

Supplementary Figure 11. *Scenario 1: (a) Modeled tsunami source off the south coast of West Java. (b) Maximum tsunami height throughout model region over duration of simulation. (c) maximum tsunami height along the south coast of Java.*

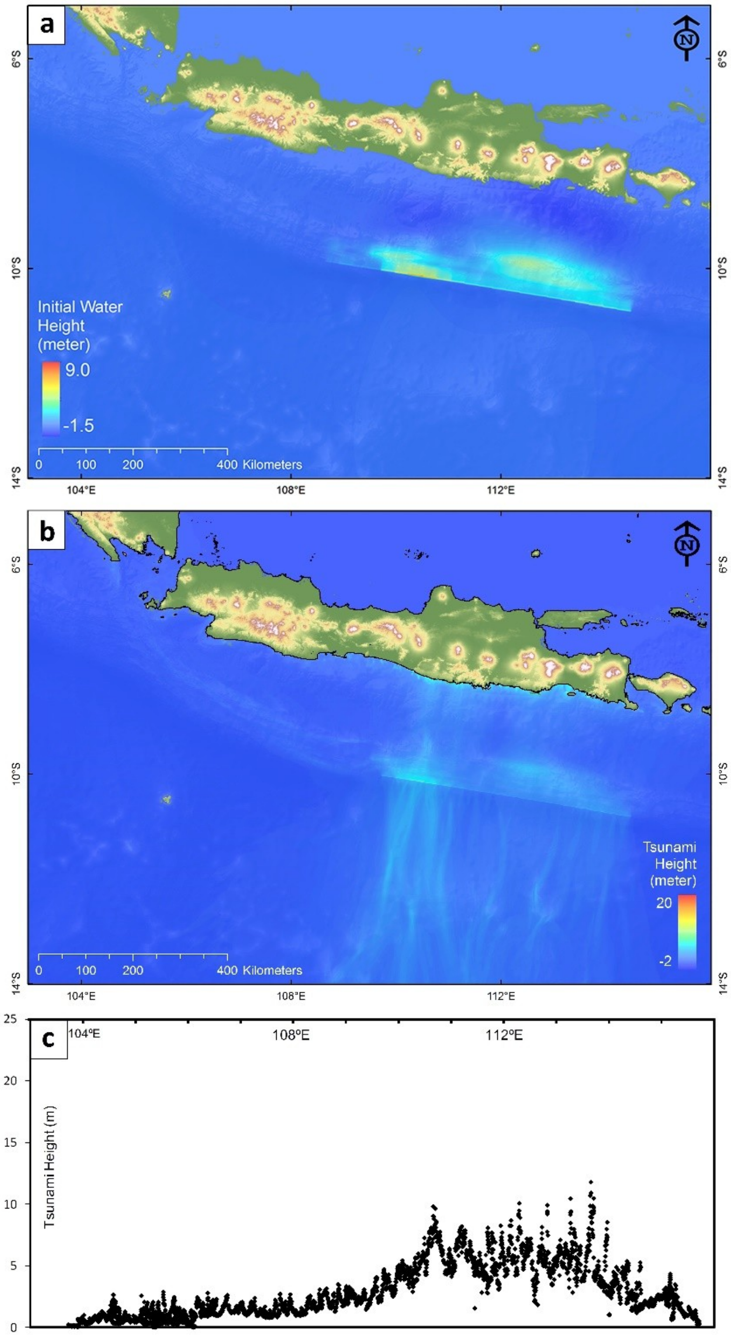

***Supplementary Figure 12**. Scenario 2: (a) Modeled tsunami source off the south coast of Central and East Java. (b) Maximum tsunami height throughout model region over duration of simulation. (c) Maximum tsunami height along the south coast of Java.*
